# Criminal responsibility evaluations: Benchmarking in different countries

**DOI:** 10.1192/j.eurpsy.2023.919

**Published:** 2023-07-19

**Authors:** H. Al-Taiar

**Affiliations:** Oxford Health NHFT, Oxford, United Kingdom

## Abstract

**Introduction:**

Forensic psychiatrists, as well as other mental health professionals, provide the legal system with clinical information and assessments concerning the offenders’ functioning, mental state and capacities at the time of the alleged offense and/or trial. These forensic assessments play a crucial role in court, influencing subsequent decision-making on sentencing, placement, or treatment of mentally disordered offenders.

**Objectives:**

Determining criminal responsibility at the time of arrest

Exploring the role of psychiatric disposals in different countries.

**Methods:**

The information was primarily gathered through written sources: peer review articles, reports, and legislation. A literature search was performed in PubMed and PsycINFO using the following keywords: criminal responsibility (reports/evaluations), pre-trial assessment,psychiatric expert, (forensic) psychiatric assessment, sanity evaluation and insanity defense. Additional articles were identified through reference lists This resulted in 36 peer review articles and nine reports or book chapters. In addition, a leading expert (i.c. psychiatrist) from every country was contacted for providing information and validating the information from this article pertaining to their country. They suggested eight more articles or book chapters. The respondents represent the authors of this article.

**Results:**

See table.

**Conclusions:**

In summary, it appears that there are distinct differences between the abovementioned countries with respect to criminal responsibility assessments. Although Canada is considered a pioneer with regard to forensic mental health, Britain, the Netherlands and Sweden appear to have a well-established system in conducting these assessments. In Sweden the system is very strict, meaning that all reports are delivered by a governmental agency with their own staff. The court orders the report from the agency and not from the experts.

**Disclosure of Interest:**

None Declared

